# Fecal Microbiota Transplant From Highly Feed Efficient Donors Affects Cecal Physiology and Microbiota in Low- and High-Feed Efficient Chickens

**DOI:** 10.3389/fmicb.2019.01576

**Published:** 2019-07-09

**Authors:** Barbara U. Metzler-Zebeli, Sina-Catherine Siegerstetter, Elizabeth Magowan, Peadar G. Lawlor, Niamh E. O′Connell, Qendrim Zebeli

**Affiliations:** ^1^Institute of Animal Nutrition and Functional Plant Compounds, Department for Farm Animals and Veterinary Public Health, University of Veterinary Medicine Vienna, Vienna, Austria; ^2^Agri-Food and Biosciences Institute, Hillsborough, United Kingdom; ^3^Teagasc, Pig Development Department, Animal & Grassland Research and Innovation Centre, Moorepark, Ireland; ^4^Institute for Global Food Security, Queen’s University Belfast, Belfast, United Kingdom

**Keywords:** broiler chicken, bacterial microbiome, fecal microbiota transplant, gut function, residual feed intake

## Abstract

Fecal microbiota transplants (FMT) may be used to improve chicken’s feed efficiency (FE) via modulation of the intestinal microbiota and microbe-host signaling. This study investigated the effect of the administration of FMT from highly feed efficient donors early in life on the jejunal and cecal microbiota, visceral organ size, intestinal morphology, permeability, and expression of genes for nutrient transporters, barrier function and innate immune response in chickens of diverging residual feed intake (RFI; a metric for FE). Chicks (*n* = 110) were inoculated with the FMT or control transplant (CT) on 1, 6, and 9 days posthatch (dph), from which 56 chickens were selected on 30 dph as the extremes in RFI, resulting in 15 low and 13 high RFI chickens receiving the FMT and 14 low and 14 high RFI chickens receiving the CT. RFI rank and FMT only caused tendencies for alterations in the jejunal microbiota and only one unclassified *Lachnospiraceae* genus in cecal digesta was indicative of high RFI. By contrast, the FMT caused clear differences in the short-chain fatty acid (SCFA) profile in the crop and cecal microbiota composition compared to the CT, which indicated alterations in amylolytic, pullulanolytic and hemicellulolytic bacteria such as *Lactobacillus*, *Dorea*, and *Ruminococcus*. Moreover, the FMT caused alterations in intestinal development as indicated by the longer duodenum and shallower crypts in the ceca. From the observed RFI-associated variation, energy-saving mechanisms and moderation of the mucosal immune response were indicated by higher jejunal permeability, shorter villi in the ileum, and enhanced cecal expression of the anti-inflammatory cytokine *IL10* in low RFI chickens. Relationships obtained from supervised multigroup data integration support that certain bacteria, including *Ruminococcocaceae-, Lactobacillus-*, and unclassified *Clostridiales*-phylotypes, and SCFA in jejunal and cecal digesta modulated expression levels of cytokines, tight-junction protein *OCLN* and nutrient transporters for glucose and SCFA uptake. In conclusion, results suggest that the intestine only played a moderate role for the RFI-associated variation of the present low and high RFI phenotypes, whereas modulating the early microbial colonization resulted in long-lasting changes in bacterial taxonomic and metabolite composition as well as in host intestinal development.

## Introduction

The microbial communities along the gastrointestinal tract of chickens play a fundamental role in gut homeostasis and development as well as host metabolism, physiology and immune functions (Borda-Molina et al., 2018), thereby modulating host feed efficiency (FE) (Schokker et al., 2015; Stanley et al., 2016). The latter relationship has prompted research to search for bacterial taxa that are associated with high FE, with the aim to use these bacteria as direct-fed microbials to modulate bird’s FE. However, FE-associated phylotypes varied greatly within and between studies (Stanley et al., 2016; Siegerstetter et al., 2017), rendering it difficult to identify unique target bacteria that may be effective across chicken populations. These divergent findings can be linked to the unique situation in commercial hatcheries where the clean hatching environment and the missing contact to adult chicken microbiota contribute to the acquisition of a gut microbiota profile that resembles the environment with high level of bird-to-bird variation (Stanley et al., 2013).

Since the establishment of the gut microbiota in chickens begins immediately post-hatch (Oakley et al., 2014; Stanley et al., 2014), stabilization of the initial colonization of the gut can be accomplished by *in ovo*-application of probiotic formulations as well as spraying or brushing of eggs and hatchlings with the gut microbiota from healthy adult birds (Rubio, 2019). These applications are successfully used on the market to transfer colonization resistance against *Salmonella* to newly hatched chickens (Rubio, 2019). Using a gut microbiota preparation or fecal microbiota transplants (FMT) allows for the consideration of the complex microbe-microbe-interactions, such as competition for nutrients and cross-feeding of metabolites, which are essential for successful colonization and are not mimicked when using single- or multiple-strain applications. Inoculating eggs with the cecal microbiota from high FE chickens the day before hatch showed to reduce bird-to-bird variation in microbiota composition, whereas this treatment was not successful to improve the FE of the recipient birds (Donaldson et al., 2017). The question remains whether this was related to the application method, reducing the survival of (strictly) anaerobic bacteria due to the exposure to the atmospheric oxygen. Previous work provided evidence for potential modification of host energy metabolism by FMT. We could recently show that a FMT administration to suckling pigs reduced body weight gain later in life (McCormack et al., 2018), whereas in chickens a FMT increased feed intake and body weight gain in female birds (Siegerstetter et al., 2018a). These findings support alterations in the “gut-brain axis” of these animals. Pathways implicated in the crosstalk between the gut microbiota and the host involve metabolites, such as short-chain fatty acids (SCFA), vagus nerve stimulation and host receptor recognition of microbiota-associated molecular patterns (MAMP) (McKenzie et al., 2017; Spielman et al., 2018). For instance, the SCFA acetate and propionate can modulate the body energy balance by reducing adipocyte lipolysis and improving leptin secretion, regulating appetite as well as enhancing the whole-body glucose homeostasis (Spielman et al., 2018). Concurrently, SCFA exert anti-inflammatory properties via G-protein activation (McKenzie et al., 2017) and straight SCFA have been associated with increased intestinal expression of tight-junction proteins, thereby improving barrier function (Morrison and Preston, 2016). The ceca are the best studied bacterial community in chickens (Oakley et al., 2014), whereas there is a dearth of information about microbe-host-interactions in the longest and for nutrient assimilation the most important gut segment, the jejunum. The present work was based on the hypothesis that a FMT prepared from excreta collected from highly feed efficient chickens may influence chicken’s early microbial colonization which will have long-lasting consequences for intestinal microbe-host signaling, development and functioning in low and highly feed efficient chickens. We further assumed that the results should enable us to distinguish RFI-related variation in intestinal physiology which is mainly influenced by the host itself. The present objective was to investigate the effect of the administration of a FMT from highly feed efficient donors early in life on the jejunal and cecal microbiota, visceral organ size, intestinal morphology, permeability, and expression of genes related to nutrient transporters, barrier function and innate immune response in broiler chickens of diverging residual feed intake (RFI; a metric for FE). Supervised sparse partial least squares (sPLS)-discriminant analysis was thereby applied to identify microbial signatures among jejunal and cecal bacteria, luminal SCFA concentrations and the host intestinal response.

## Materials and Methods

### Animals and Diets

The samples used in this study originated from the same chickens from which data were presented in Siegerstetter et al. (2018a). In brief, day-old Cobb 500 broiler chicks of both sexes were used in two consecutive replicate batches (batch 1, *n* = 54; and batch 2, *n* = 56; equal numbers of males and females per batch), with 1 more female and 1 more male in batch 2 compared to batch 1. Housing and environmental conditions have been previously described (Siegerstetter et al., 2018a). Chicks of the same sex were group-housed in the first 8 dph (*n* = 5–6 chicks/cage). The housing and environmental conditions were previously described (Metzler-Zebeli et al., 2016b). From 9 dph until the end of the experiment (33–37 dph), chicks were kept individually in metabolism cages to determine their individual feed intake. All chickens had *ad libitum* access to demineralized water and starter (1–8 dph), grower (9–20 dph), and finisher (21–37 dph) corn-soybean meal based diets (Siegerstetter et al., 2018a) which were free of antimicrobials and coccidiostats. For both treatment groups, fresh feed was provided at 9:00 am and feeders were re-filled with feed at 3:00 pm to ensure *ad libitum* access to feed.

### Preparation of the FMT and Inoculation of Day-Old Chickens

The preparation of the FMT and inoculation procedure have been already described in detail in Siegerstetter et al. (2018a). Low RFI chickens from a previous chicken experiment (Siegerstetter et al., 2018b) were monitored on 30 dph and a sufficient amount of freshly dropped excreta could be collected from four females and two males to prepare the FMT stock for each donor separately. Each fecal dropping was immediately processed under anaerobic conditions and was continuously kept on ice throughout the procedure. The white portion of the excreta was removed, twice the amount of sterile phosphate buffered saline (PBS) was added, and the slurry was homogenized and centrifuged at low speed (800 × *g* for 3 min at 4°C; Eppendorf Centrifuge 5810 R, Eppendorf, Hamburg, Germany) to separate undigested feed and particulate material from the microbial fraction. To ensure microbial survival during storage (−80°C), the supernatant from each donor was mixed with sterile glycerol (10% vol) and kept on ice for 60 min to allow the glycerol to penetrate the bacterial cells. Finally, the fecal suspension was aliquoted. On the inoculation days, one aliquot of the fecal suspension from each low RFI female and male chicken was thawed on ice and equal volumes of the single suspensions were combined to form the FMT stock. Anaerobic and aerobic culturing and quantitative PCR (qPCR) was used to estimate the bacterial numbers in the prepared FMT stock before the start of the chicken experiment and on each inoculation day (Siegerstetter et al., 2018a). Upon arrival and on 6 and 9 dph, chickens were either inoculated with 100 μl of the FMT [10^4^ colony forming units (CFU)] or the control transplant (CT) which consisted of sterile PBS. Chickens housed together received the same transplant. The transplant was orally administered at the back of the tongue using a syringe and chicks were supervised that they swallowed. On 6 and 9 dph, feed was withheld for 15 min before and after the administration.

### Determination of FE

The individual feed intake per bird was recorded on 9, 14, 21, 28, and 30 dph. Feed refusals were collected daily before morning feeding, and feed spills were collected weekly. Chickens were weighed on 1, 7, 9, 14, 21, 28, and 30 dph. The RFI as metric for FE was determined between 9 to 30 dph. Chicken’s RFI as the residuals over the test period (9–30 dph) was based on the data for total feed intake (TFI), metabolic mid-test BW (MMW), and total body weight gain (TBWG) using a non-linear mixed model (SAS Stat Inc., version 9.4; Cary, NC, United States; Metzler-Zebeli et al., 2016a). In each replicate batch, separately for females and males and balanced for batch, the chickens with the lowest RFI (good FE) and highest RFI (poor FE) values in each treatment group were selected to be sampled. This resulted in 15 low (females, *n* = 8; and males, *n* = 7) and 13 high (females, *n* = 7; and males, *n* = 6) RFI chickens receiving the FMT and 14 low (*n* = 7/sex) and 14 high (*n* = 7/sex) RFI chickens receiving the CT.

### Intestinal Sampling

The body weight was determined before chickens were euthanized with an overdose of Thiopental (50–100 mg/kg, medicamentum pharma GmbH, Allerheiligen im Mürztal, Austria) by i.v. injection into the caudal tibial vein between 33 and 37 dph. The abdominal cavity was opened and the visceral organs were removed (Metzler-Zebeli et al., 2018b). Visceral organ weight and length of the individual intestinal segments were measured and expressed per kg of body weight to account for differences in individual body weight. Luminal digesta was collected and homogenized using a spatula before snap-freezing in liquid nitrogen and long-term storage at −80°C for microbiota analysis or short-term storage on ice and storage at −20°C for SCFA analysis. Pieces of the intestinal tube (1 cm) for morphometric measurements were collected at the Meckel’s diverticulum, from the first centimeter of the proximal ileum, and proximal to the blind end of the ceca. Those pieces were cleaned with phosphate-buffered saline and fixed in neutral-buffered (pH 7.0) formalin (4% vol/vol). A 20-cm tissue tube piece for the electrophysiological (Ussing chamber) experiment was removed distal to the Meckel’s diverticulum, immediately transferred into ice-cold and pre-gassed [carbogen gas (95% O_2_–5% CO_2_)] transport buffer (Metzler-Zebeli et al., 2018b), and transported to the laboratory within 10 min after the death of the animal. The jejunum was opened at the mesenterium and the ceca longitudinally, washed in neutral-buffered saline and blotted dry with paper tissue. The mucosa was scraped off from the jejunum between the Meckel’s Diverticulum and 35 cm toward the duodenum as well as from both ceca using a glass slide. The mucosa scrapings were immediately snap-frozen in liquid nitrogen and aliquots were stored at −80°C for RNA isolation.

### DNA Extraction and 16S rRNA Gene Sequencing

Total DNA was extracted from 300 μl of the prepared FMT stocks, as well as from 250 mg of ileal (*n* = 55) and cecal samples (*n* = 56) using a modified protocol of the PowerSoil DNA isolation kit (MoBio Laboratories Inc., Carlsbad, CA, United States; Metzler-Zebeli et al., 2016b). The DNA concentration was quantified using the Qubit 2.0 Fluorometer (Life Technologies, Carlsbad, CA, United States) with the Qubit dsDNA HS Assay Kit (Life Technologies). An aliquot of each DNA sample was sent to a commercial provider (Microsynth AG, Balgach, Switzerland), where the V3–V5 hypervariable region of the 16S rRNA gene was amplified using the primers 357F-HMP (5′-CCTACGGGAGGCAGCAG-3′) and 926R-HMP (5′-CCGTCAATTCMTTTRAGT-3′) to generate an approximate amplicon size of 570 bp (Peterson et al., 2009) as previously described (Siegerstetter et al., 2018a,b). The KAPA HiFi HotStart PCR Kit (Roche, Baden, Switzerland) was used, which includes a high-fidelity DNA polymerase. Libraries were constructed by ligating sequencing adapters and indices onto purified PCR products using the Nextera XT sample preparation kit (Illumina Inc., San Diego, CA, United States). Equimolar amounts for each library were pooled and sequenced on an Illumina MiSeq Personal Sequencer using a 300 bp read length paired-end protocol, whereby all sample libraries were sequenced in the same sequencing run. The obtained FASTQ files were de-multiplexed, trimmed of Illumina adaptor residuals using cutadapt (version 1.8.1)^1^ and the overlapping paired-end reads were stitched using Fast Length Adjustment of SHort reads (FLASH, version 1.2.11)^2^ by Microsynth (Siegerstetter et al., 2017). Raw sequencing data are available in NCBI’s BioProject SRA database (PRJNA392215 and PRJNA 529338).

Sequence data were analyzed with the Quantitative Insights Into Microbial Ecology (QIIME) package version 1.9.1 (Caporaso et al., 2010) as previously described (Metzler-Zebeli et al., 2019). The sequences for the eight FMT samples (Siegerstetter et al., 2018a) were re-analyzed together with the current dataset. After quality trimming of the stitched reads using a quality threshold of *q* > 20, the UCHIME method using the 64-bit version of USEARCH (Edgar, 2010; Edgar et al., 2011) and the GOLD database (drive5.com) were used to screen for and exclude chimeric sequences. Open-reference operational taxonomic unit (OTU) picking was done at 97% similarity level using UCLUST (Edgar, 2010) and the Greengenes database as reference template (version 13_8) (DeSantis et al., 2006). Alpha-diversity measurements were performed by means of the “vegan” R package (version 2.5.2) (Oksanen et al., 2018). For β-diversity analysis, dissimilarity matrices (Bray–Curtis) derived from OTU data were calculated with PERMANOVA using the “adonis2” function and visualized in two-dimensional non-metric multidimensional scaling (NMDS) ordination plots obtained with the “metaMDS” function in the vegan R package. To test for differences in relative bacterial abundances, only taxa appearing in at least 50% of the samples were considered. The raw read counts from the OTU abundance table were collapsed at taxonomic rank and compositionally normalized such that each sample sums to 1.

### Histo-Morphology

Histo-morphological measurements were performed as previously described (Metzler-Zebeli et al., 2018b). After fixation, intestinal tube pieces were dehydrated in ethanol, cleared in xylene and embedded in paraffin. Three discontinuous 3–4 μm-thick sections per intestinal site were routinely stained with hematoxylin and eosin. The Leica DM2000 light microscope (Leica Microsystems, Wetzlar, Germany) fitted with a digital camera (Leica DFC425C) and the Leica Application Suite V3.7 software was used to take pictures which were analyzed with ImageJ software (Version 1.47; National Institutes of Health, Maryland, United States). Fifteen intact well-oriented, crypt-villus units were selected, with the criterion for villus selection based on the presence of intact lamina propria. Villus height and width were measured at 4-times magnification and crypt depth at 10-times magnification. The circular and longitudinal muscular layers were measured. Goblet cells were counted per 250 μm of villus or crypt epithelium, 15 replicates per gut site at 10-times magnification, whereas intraepithelial lymphocytes were counted per 400 μm villus epithelium, 12 replicates per gut site at 20-times magnification. Goblet cell counts and intraepithelial lymphocytes were presented per villus-crypt unit.

### Intestinal Electrophysiology

Differences in intestinal electrophysiological parameters and permeability marker flux rates were evaluated for four chickens per sampling day (Metzler-Zebeli et al., 2017, 2018b). This resulted in five observations per RFI and inoculum group. After opening at the mesenterium and rinsing with transport butter, the outer serosal layers were stripped and three consecutive pieces were cut from the proximal 10 cm of the jejunal tube, mounted in Ussing chambers (exposed area of 0.91 cm^2^) and incubated in a total volume of 10 mL serosal and mucosal buffer solution (pH 7.4, 38°C) (Metzler-Zebeli et al., 2018b). Continuous gassing with carbogen using a gas lift was provided on the mucosal and the serosal sides for oxygenation of the tissues and circulation of the buffer. Two pairs of dual channel current and voltage Ag-AgCl electrodes connected via 3% agar bridges filled with 3 M potassium chloride were used to continuously record the potential difference (mV), short-circuit current (*I*_sc_, μA/cm^2^) and transepithelial resistance (Ω × cm^2^) with a microprocessor-based voltage-clamp device and software (version 9.10; Mussler, Microclamp, Aachen, Germany). The tissue was alternatively pulsed with a positive or negative pulse of 20 μA and 100 ms duration. After an equilibration period of 20 min under open-circuit conditions, the tissue was short-circuited by clamping the voltage to zero. After recording electrophysiological measurements for 5 min, fluorescein 5(6)-isothiocyanate (FITC; 389.38 g/mol; Sigma-Aldrich, Schnelldorf, Austria) and horse-radish peroxidase (HRP; 44,000 g/mol; Carl Roth GmbH + Co. KG, Karlsruhe, Germany) were added to final concentrations of 0.1 mM and 1.8 μM to the mucosal side, respectively, to assess the mucosal-to-serosal flux (Metzler-Zebeli et al., 2018b). The glucose absorptive tissue response was studied by adding glucose to a final concentration of 10 mmol/L to the buffer at the mucosal side at 45 min after short-circuiting the tissue (Metzler-Zebeli et al., 2017). The chemical effect on glucose transporter function was measured by comparing the *I*_sc_ and *R*_T_ for 1 min before glucose was added to the peak current and resistance response of the exposed tissue (ΔI_sc_ and ΔR_T_) obtained within 2 min after the addition of glucose.

### Mucosal Gene Expression

Total RNA was isolated from jejunal and cecal mucosa samples using mechanical homogenization (FastPrep-24 instrument; MP Biomedicals, Santa Ana, CA, United States) and the RNeasy Mini Kit (Qiagen, Hilden, Germany) (Metzler-Zebeli et al., 2018a). The RNA isolates were treated with DNase I (RNA Clean & Concentrator-5 Kit, Zymo Research, Irvine, United States) before transcribing 2 μg of total RNA into single stranded cDNA using the High Capacity Reverse Transcription Kit (Life Technologies Foster City, United States). The quality of the isolated RNA was verified using the Agilent 2100 Bioanalyzer (Agilent Technologies, Santa Clara, United States), showing RNA integrity numbers between 8 and 10.

Primers for target genes and housekeeping genes (HKG) as well as amplification conditions used were recently published by our group (Metzler-Zebeli et al., 2019). Amplifications were performed on the CFX96 Touch Real-Time PCR Detection System (Bio-Rad Laboratories Ges.m.b.H, Wien, Austria) using the following conditions: 95°C for 5 min, followed by 95°C for 10 s, 60°C for 30 s, and 72°C for 30 s for 40 cycles, followed by the generation of dissociation curves. Each 20 μL reaction consisted of 50 ng cDNA, 10 μL Fast Plus Eva Green master mix with low ROX (Biotium, Hayward, CA, United States), 100 nM each of forward and reverse primers, and DEPC-treated water in a 96-well plate (VWR, Vienna, Austria). All reactions including negative controls and reverse transcription controls (RT minus) to control for residual DNA contamination were run in duplicate. Of the six tested HKG, *ACTB, B2M*, and *GAPDH* were most stably expressed, which was analyzed using NormFinder (Andersen et al., 2004) and BestKeeper (Pfaffl et al., 2004) based analysis identified *ACTB, B2M*, and *GAPDH* as the most stably expressed HKG. Their geometric mean expression level was used for normalization of target gene expression levels. For this, the mean Cq values of the identified HKG were subtracted from the Cq of the target genes to determine ΔCq values. Relative gene expression was calculated relative to the chicken with the highest expression of the respective gene using the 2^−ΔΔ*Cq*^ method. Amplification efficiencies (*E* = 10^(−1/*slope*)−1^) of all primer sets, ranging from 90.6 to 107.0% (*R*^2^ = 0.98–1.00) were obtained by a fivefold serial dilution of pooled samples.

### Short-Chain Fatty Acid Analysis

For the analysis of SCFA (acetate, propionate, butyrate, iso-butyrate, valerate, iso-valerate and caproate), 1 g digesta from crop, jejunum, ileum and ceca was mixed with 0.2 ml of 25% metaphosphoric acid, 1 ml of double distilled water and 200 μl of internal standard (4-methyl-valeric acid; Sigma-Aldrich, Vienna, Austria), and centrifuged at 3148 × *g* for 10 min (Centrifuge 5810 R, Eppendorf, Hamburg, Germany) (Metzler-Zebeli et al., 2019). The supernatant was centrifuged at 15,000 × *g* for 25 min (Centrifuge 5424, Eppendorf) and the clear supernatant was analyzed using gas chromatography.

### Statistical Analyses

Key feature identification was performed using multigroup supervised DIABLO N-integration by means of the package “mixOmics” (version 6.3.2) (Rohart et al., 2017) in R studio (version 1.0.136). Horizontal sparse partial least squares-discriminant analysis (sPLS-DA) was used to integrate the datasets of relative abundances of OTUs, SCFA and mucosal expression levels of target genes in the jejunum and ceca to classify and select key features from each dataset. The tuning of sPLS-DA parameters was performed to determine the main OTUs, SCFA, and mucosal expression levels of genes that enable discrimination of treatments groups with the lowest possible error rate. We tuned the number of retained variables to one-fourth of all parameters per dataset, selecting 8 OTUs (>0.05% relative abundance), five genes and two SCFA for the jejunum and 20 OTUs (>0.05% relative abundance), five genes, two SCFA for the ceca for components 1 and 2, respectively. The sPLS-DA results were visualized as circos plots showing the strongest positive and negative Pearson’s correlations (|r| > 0.7) between most discriminant OTUs, SCFA and mucosal expression levels of genes for each subset of data and identified features. Additionally, sPLS and relevance network analysis were performed using “mixOmics” (Lê Cao et al., 2016; Rohart et al., 2017) to integrate data of OTUs (0.01% of all reads) in jejunal and cecal digesta with results for TFI, TBWG and RFI, cecal morphology (crypt depth, goblet cell counts and lymphocyte counts), weight and length.

After analyzing for normality using the Shapiro-Wilk test (SAS Stat Inc., version 9.4; Cary, NC, United States), FE parameters, data for bacterial taxonomy, SCFA, visceral organ size, gut structure and function were subjected to ANOVA using the MIXED procedure in SAS. In regard to the microbiome data, only the bacterial taxa comprising a relative abundance > 0.01% of all reads across both sexes were analyzed by ANOVA. The fixed effects of batch, sex, FMT, RFI, and the two-way-interaction FMT × RFI were considered in the main model. Batch was considered as random effect in the final model. Chicken nested within sex and sampling day was the experimental unit. Degrees of freedom were approximated using the Kenward–Roger method. Differences among least squares means were computed using the pdiff statement. Differences were considered significant if *P* ≤ 0.05 and as trend if 0.05 < *P* ≤ 0.10. Descriptive statistics on bacterial composition of the FMT were performed using the MEANS procedure in SAS.

## Results

### Performance and Feed Efficiency

Chickens with extremely low and high RFI values were selected in both inocula groups to discriminate between their gut microbiota, structure and function. As a result, chickens in both inocula groups had largely contrasting RFI values as described in our sister article (Siegerstetter et al., 2018a; Supplementary Table S1). In brief, the RFI was 289 g lower in low (good feed efficient) compared to high (poor feed efficient) RFI chickens across both sexes (*P* < 0.001).

### FMT- and FE-Associated Bacterial Microbiome in Jejunal and Cecal Digesta

After quality control and chimera check, a total of 2,551,214 sequencing reads with a mean of 21,439 sequences per sample were obtained for the 111 gut and 8 FMT stock samples (mean read length 537 bp). The FMT consisted predominantly of four OTUs, *Escherichia*-OTU1, -OTU22 and -OTU25 (71.1%) and *Turicibacter*-OTU4 (21.2%; Supplementary Figure S1).

Bray–Curtis-derived dissimilarity matrices (PERMANOVA) for jejunal and cecal bacterial composition indicated that the FMT and RFI rank did not affect (*P* > 0.10) the β-diversity structure. This was supported by similar diversity and species richness among treatment groups (Supplementary Table S2). However, a significant separation (Bray-Curtis) was detected between the bacterial composition of the FMT and the jejunal and cecal communities (*P* < 0.05; Supplementary Figure S2).

Taxonomic assessment of the bacterial microbiota in jejunal and cecal digesta showed that both RFI rank and FMT only caused tendencies for alterations in the jejunal microbiota (Supplementary Tables S3, S4), whereas clear differences in relative bacterial abundances were detectable for the cecal microbiota of chickens receiving the FMT compared to the CT (Table 1). In jejunal digesta, the FMT tended (*P* < 0.10) to promote *Blautia* and *Phenylobacterium* (Supplementary Table S4). Also, in the jejunum, high RFI was associated with trends for higher abundances of *Turicibacter*, *Sphingomonas*, an unclassified *0319-6G20* genus belonging to *Myxococcales* and *Phenylobacterium* compared to low RFI. Similar to jejunal digesta, the dominant phyla were equally abundant among treatment groups in the ceca. Only *Tenericutes* (relative abundance 0.80% of all reads; Supplementary Table S3) were 1.9-fold more abundant in low versus high RFI chickens but only in those birds receiving the CT as indicated by the FMT × RFI interaction (*P* < 0.05). This increase was mainly due to the increased abundance of an unclassified genus within the order *RF39* in low RFI chickens receiving the CT (*P* = 0.045; Table 1). In total, five genera were and one tended to be affected by the FMT in cecal digesta, with *Lactobacillus*, unclassified genera within *Bacillaceae* and *Ruminococcaceae* (*P* < 0.05) being less abundant, and *Ruminococcus* (*P* < 0.10), an unclassified *Lachnospiraceae* genus and *Dorea* (*P* < 0.05) being more abundant in chickens receiving the FMT compared to those receiving the CT. The same unclassified *Lachnospiraceae* genus as in the jejunum was associated with high RFI in cecal digesta (Table 1 and Supplementary Table S4).

**Table 1:** Differences in relative abundance (%) of most abundant bacterial genera present in cecal digesta of low and high residual feed intake (RFI) broiler chickens receiving either a fecal microbiota transplant (FMT) or a control transplant (CT).

	FMT		CT			*P* value		
Genus	Low RFI	High RFI	Low RFI	High RFI	SEM	FMT	RFI	FMT × RFI
g_*Lactobacillus*	0.68	0.35	1.23	0.90	0.254	0.036	0.195	0.995
g_*Ruminococcus*	3.11	3.70	2.57	2.94	0.380	0.093	0.215	0.772
g_[*Ruminococcus*]	2.42	2.79	1.30	1.79	0.460	0.025	0.352	0.904
o_*RF39*;f_;g_	0.68	1.01	1.15	0.40	0.264	0.788	0.429	0.045
g_*Dorea*	0.27	0.24	0.16	0.18	0.042	0.047	0.912	0.528
f_*Lachnospiraceae*;f_;g_	0.18	0.27	0.15	0.21	0.035	0.190	0.032	0.520
f_*Bacillaceae*;g_	0.016	0.022	0.098	0.035	0.023	0.050	0.229	0.146
f_*Ruminococcaceae*;g_	0.030	0.027	0.048	0.049	0.008	0.012	0.889	0.833

*Data are presented as least-square means and pooled SEM. Low RFI FMT females, n = 8; low RFI FMT males, n = 7; high RFI FMT females, n = 7; high-RFI FMT males, n = 6; low RFI CT females, n = 7; low-RFI CT males, n = 7; high-RFI CT females, n = 7; high-RFI CT males, n = 7.*

### FMT- and RFI-Associated Effects on Jejunal and Cecal Fermentation Acids

The FMT decreased the concentration of total SCFA (*P* < 0.10) and acetate (*P* < 0.05) in the crop by 26.7 and 28.5%, respectively, compared to the CT (Table 2). By contrast, minor SCFA, i.e., isobutyrate, valerate (*P* < 0.10) and isovalerate (*P* < 0.05) increased with the FMT compared to the CT in the crop. Moreover, there was a trend (*P* < 0.10) for less isobutyrate in jejunal digesta and a 46.4%-enhanced (*P* < 0.05) propionate concentration in cecal digesta with the FMT compared to the CT. Jejunal digesta were more concentrated in total SCFA in high RFI chickens that received the FMT compared to their low RFI counterparts, whereas no difference between RFI ranks was found for the CT as indicated by the FMT × RFI interaction (*P* < 0.05). This was partly due to the trend (*P* < 0.10) for more acetate in jejunal digesta of high versus low RFI chickens. Results for the changes in molar proportions among the four chicken groups are presented in Supplementary Table S5, supporting the changes observed on concentration basis.

**Table 2:** Differences in concentrations of short-chain fatty acids (SCFA) in crop, jejunal, ileal, and cecal digesta of low and high residual feed intake (RFI) broiler chickens receiving either a fecal microbiota transplant (FMT) or a control transplant (CT).

	FMT		CT			*P* value		
Item	Low RFI	High RFI	Low RFI	High RFI	SEM	FMT	RFI	FMT × RFI
**Crop**								
Total SCFA	43.12	44.71	60.24	59.55	7.908	0.052	0.955	0.885
Acetate	38.36	38.68	54.21	53.55	7.146	0.039	0.982	0.945
Propionate	0.68	1.06	0.60	0.51	0.215	0.149	0.509	0.282
Isobutyrate	3.12	3.35	4.44	4.60	0.740	0.092	0.789	0.961
Butyrate	0.49	0.71	0.60	0.54	0.142	0.820	0.573	0.326
Isovalerate	0.08	0.14	0.02	0.02	0.037	0.023	0.410	0.354
Valerate	0.13	0.27	0.08	0.08	0.060	0.063	0.238	0.230
Caproate	0.27	0.48	0.28	0.24	0.085	0.191	0.312	0.133
**Jejunum**								
Total SCFA	47.88^b^	61.73^a^	54.95^a,b^	54.72^a,b^	3.236	0.994	0.041	0.036
Acetate	44.14	51.37	48.08	51.61	2.845	0.476	0.065	0.518
Propionate	0.27	0.28	0.28	0.24	0.054	0.735	0.759	0.624
Isobutyrate	4.23	5.49	5.57	6.26	0.584	0.084	0.101	0.628
Butyrate	0.16	0.18	0.13	0.13	0.042	0.297	0.830	0.776
Isovalerate	0.23	0.21	0.18	0.23	0.072	0.828	0.847	0.645
Valerate	0.01	0.01	0.01	0.00	0.009	0.515	0.824	0.598
Caproate	0.19	0.15	0.10	0.15	0.049	0.382	0.951	0.322
**Ileum**								
Total SCFA	41.18	44.88	38.46	38.80	3.060	0.158	0.514	0.586
Acetate	37.72	40.94	35.33	35.41	2.758	0.159	0.555	0.574
Propionate	0.29	0.38	0.29	0.33	0.049	0.594	0.228	0.597
Isobutyrate	2.89	3.20	2.61	2.79	0.290	0.238	0.402	0.818
Butyrate	0.03	0.03	0.04	0.03	0.020	0.882	0.795	0.692
Isovalerate	0.23	0.27	0.14	0.20	0.059	0.166	0.408	0.888
Valerate	ND	ND	ND	ND	ND	ND	ND	ND
Caproate	0.01	0.05	0.04	0.04	0.021	0.600	0.345	0.268
**Ceca**								
Total SCFA	147.00	155.91	126.56	139.07	13.710	0.186	0.460	0.896
Acetate	118.51	128.07	103.43	113.88	11.620	0.220	0.415	0.970
Propionate	7.55	9.24	5.72	5.75	0.985	0.012	0.409	0.408
Isobutyrate	1.06	0.77	0.99	1.26	0.167	0.214	0.958	0.107
Butyrate	16.83	15.03	13.62	14.54	2.771	0.510	0.880	0.627
Isovalerate	0.99	0.77	0.93	1.28	0.204	0.278	0.759	0.176
Valerate	1.54	1.40	1.38	1.51	0.207	0.918	0.973	0.522
Caproate	0.53	0.63	0.49	0.85	0.164	0.571	0.190	0.440

*Data are presented as least-square means and pooled SEM. Low RFI FMT females, n = 8; low RFI FMT males, n = 7; high RFI FMT females, n = 7; high-RFI FMT males, n = 6; low RFI CT females, n = 7; low-RFI CT males, n = 7; high-RFI CT females, n = 7; high-RFI CT males, n = 7. ND, not detected. ^a,b^Different superscripts within a row indicate significant difference (P ≤ 0.05).*

### FMT- and RFI-Associated Effects on Visceral Organ Size and Gut Histo-Morphology

The FMT increased the length of the duodenum (*P* < 0.05) and, as trend, of the ceca (*P* < 0.10) by 8.3 and 7.4%, respectively, compared to the CT (Supplementary Table S6). Moreover, the ceca of low RFI chickens weighed 10% more than those of high RFI chickens (*P* < 0.05). The trend (*P* < 0.10) for a FMT × RFI interaction indicated a higher gizzard weight in high versus low RFI chickens receiving the FMT but not in birds receiving the CT. In the ileum, the villus height and surface were smaller by 8.4 and 15.0%, respectively, in low RFI compared to high RFI birds (*P* < 0.05) but unaffected by the FMT (Supplementary Table S7). By contrast, the FMT reduced the crypt depth in the ceca by 14.6% (*P* < 0.05) and tended (*P* < 0.10) to reduce the circular muscle and goblet cell number by 9.7 and 24.7% compared to the CT, respectively.

### FMT- and RFI-Associated Effects on Jejunal Electrophysiology

Low RFI chickens tended (*P* < 0.10) to have a greater mucosal-to-serosal-flux rate of the small molecular weight marker HRP compared to high RFI birds (Table 3). Moreover, the jejunal tissue of low RFI chickens had a higher basal G_T_ and showed a greater change in G_T_ as response to the mucosally added glucose compared to high RFI animals (*P* ≤ 0.05).

**Table 3:** Differences in mucosal permeability and response to luminal glucose addition in distal jejunum of low and high residual feed intake (RFI) broiler chickens receiving either a fecal microbiota transplant (FMT), or a control transplant (CT).

	FMT		CT			*P* value		
Item	Low RFI	High RFI	Low RFI	High RFI	SEM	FMT	RFI	FMT × RFI
**Average electrophysiological parameters**								
FITC (nmol/cm^2^ × h)^1^	0.015	0.191	0.005	0.008	0.0883	0.283	0.320	0.335
HRP (pmol/cm^2^ × h)^1^	0.015	0.005	0.021	0.007	0.0062	0.489	0.057	0.729
**Glucose response^2^**								
Basal *I*_sc_ (μA/cm^2^)	−4.28	−3.96	−2.65	−0.69	1.872	0.200	0.548	0.665
ΔI_sc_	2.97	2.28	2.69	2.54	0.202	0.955	0.047	0.184
Basal *R*_T_ (W/cm^2^)	2.29	2.03	1.92	2.18	0.435	0.795	1.000	0.553
ΔR_T_	0.09	0.06	0.08	0.08	0.008	0.843	0.050	0.121

*Data are presented as least-square means and pooled SEM. Low RFI FMT females, n = 8; low RFI FMT males, n = 7; high RFI FMT females, n = 7; high-RFI FMT males, n = 6; low RFI CT females, n = 7; low-RFI CT males, n = 7; high-RFI CT females, n = 7; high-RFI CT males, n = 7. ^1^FITC, fluorescein 5(6)-isothiocyanate; HRP, horseradish peroxidase. ^2^Glucose addition to a final chamber concentration of 5 mmol/l; ΔI_sc_ is the difference between the maximal I_sc_ value obtained from 2 min after glucose addition and the basal value determined 1 min before glucose addition; ΔR_T_ is the difference between the basal GT 1 min before glucose addition and the R_T_ value obtained from 2 min after glucose addition.*

### FMT- and RFI-Associated Effects on Jejunal and Cecal Gene Expression Levels

Low RFI chickens tended (*P* < 0.10) to have lower expression levels of *NFKB* in the jejunum compared to their high RFI counterparts (Table 4). The trend (*P* < 0.10) for a FMT × RFI interaction for *MUC2* indicated higher expression levels at the jejunal mucosa in high versus low RFI birds receiving the FMT but not in those receiving the CT. By contrast, expression levels of *SMCT* tended (*P* < 0.10) to be higher in low versus high RFI birds but only in those receiving the CT. At the cecal mucosa, there was a trend (*P* < 0.10) for a reduced *IL6* expression with the FMT compared to the CT. Low RFI chickens had greater expression levels of *IL10* (*P* < 0.05) and *IL1B* (*P* < 0.10), respectively, and tended to have a lower expression of *CLDN1* at the cecal mucosa compared to high RFI birds (*P* < 0.10). By contrast, cecal *MCT1* expression levels (*P* < 0.05) were higher in low versus high RFI chickens but only in birds receiving the FMT as indicated by the FMT × RFI interaction (*P* < 0.05).

**Table 4:** Differences in expression levels of nutrient transporter, barrier, and innate immune genes at the jejunal and cecal mucosa of low and high residual feed intake (RFI) broiler chickens receiving either a fecal microbiota transplant (FMT) or a control transplant (CT).

	FMT		CT			*P* value		
Gene of interest	Low RFI	High RFI	Low RFI	High RFI	SEM	FMT	RFI	FMT × RFI
**Jejunum**								
*IAP*	0.365	0.456	0.434	0.422	0.048	0.715	0.416	0.284
*MUC1*	0.127	0.120	0.056	0.088	0.051	0.318	0.809	0.700
*MUC2*	0.312	0.469	0.353	0.346	0.045	0.369	0.100	0.074
*CLDN1*	0.246	0.314	0.286	0.276	0.051	0.983	0.573	0.444
*CLDN5*	0.128	0.139	0.083	0.104	0.044	0.362	0.719	0.905
*OCLN*	0.378	0.394	0.394	0.401	0.021	0.569	0.579	0.846
*ZO1*	0.337	0.336	0.288	0.331	0.037	0.467	0.585	0.556
*SGLT1*	0.316	0.380	0.419	0.404	0.037	0.091	0.504	0.288
*GLUT2*	0.419	0.437	0.440	0.402	0.048	0.879	0.835	0.562
*MCT1*	0.129	0.127	0.120	0.125	0.017	0.750	0.942	0.853
*SMCT*	0.082	0.095	0.133	0.042	0.030	0.974	0.195	0.085
*IL1B*	0.056	0.040	0.055	0.059	0.009	0.309	0.486	0.273
*IL6*	0.253	0.265	0.319	0.343	0.056	0.205	0.747	0.915
*IL8*	0.005	0.007	0.010	0.006	0.003	0.500	0.795	0.312
*IL10*	0.010	0.013	0.017	0.012	0.005	0.478	0.894	0.385
*TGFB1*	0.169	0.123	0.103	0.118	0.037	0.347	0.687	0.411
*TNFA*	0.058	0.067	0.069	0.066	0.004	0.298	0.518	0.188
*NFKB*	0.164	0.211	0.197	0.215	0.018	0.295	0.078	0.431
*TLR2*	0.099	0.039	0.032	0.033	0.036	0.311	0.411	0.407
*TLR4*	0.308	0.270	0.314	0.343	0.038	0.307	0.909	0.385
**Ceca**								
*IAP*	0.127	0.104	0.119	0.119	0.014	0.802	0.409	0.401
*MUC1*	0.226	0.162	0.189	0.254	0.043	0.534	0.994	0.148
*MUC2*	0.035	0.072	0.021	0.021	0.026	0.221	0.480	0.498
*CLDN1*	0.299	0.317	0.271	0.340	0.023	0.924	0.070	0.273
*CLDN5*	0.190	0.132	0.166	0.185	0.020	0.466	0.342	0.063
*OCLN*	0.468	0.531	0.536	0.479	0.056	0.889	0.948	0.290
*ZO1*	0.494	0.448	0.452	0.495	0.042	0.948	0.971	0.291
*SGLT1*	0.015	0.038	0.012	0.011	0.015	0.314	0.455	0.416
*MCT1*	0.556^a^	0.360^b^	0.458^a,b^	0.495^a,b^	0.055	0.743	0.154	0.039
*SMCT*	0.090	0.057	0.005	0.001	0.056	0.214	0.744	0.796
*IL1B*	0.364	0.237	0.366	0.326	0.045	0.314	0.068	0.336
*IL6*	0.272	0.199	0.309	0.292	0.036	0.079	0.222	0.435
*IL8*	0.121	0.042	0.066	0.034	0.035	0.378	0.124	0.501
*IL10*	0.324	0.142	0.215	0.161	0.219	0.415	0.036	0.245
*TGFB1*	0.324	0.316	0.310	0.293	0.033	0.580	0.710	0.887
*TNFA*	0.071	0.127	0.062	0.065	0.034	0.309	0.393	0.448
*NFKB*	0.530	0.413	0.463	0.402	0.065	0.545	0.177	0.667
*TLR2*	0.099	0.087	0.104	0.104	0.007	0.131	0.389	0.373
*TLR4*	0.569	0.716	0.553	0.549	0.140	0.515	0.610	0.591

*Data are presented as least-square means and pooled SEM. Low RFI FMT females, n = 8; low RFI FMT males, n = 7; high RFI FMT females, n = 7; high-RFI FMT males, n = 6; low RFI CT females, n = 7; low-RFI CT males, n = 7; high-RFI CT females, n = 7; high-RFI CT males, n = 7. ^a,b^Different superscripts within a row indicate significant difference (P ≤ 0.05).*

### Relationships Among OTUs, SCFA, and Host Mucosal Gene Expression

**FIGURE 1:**
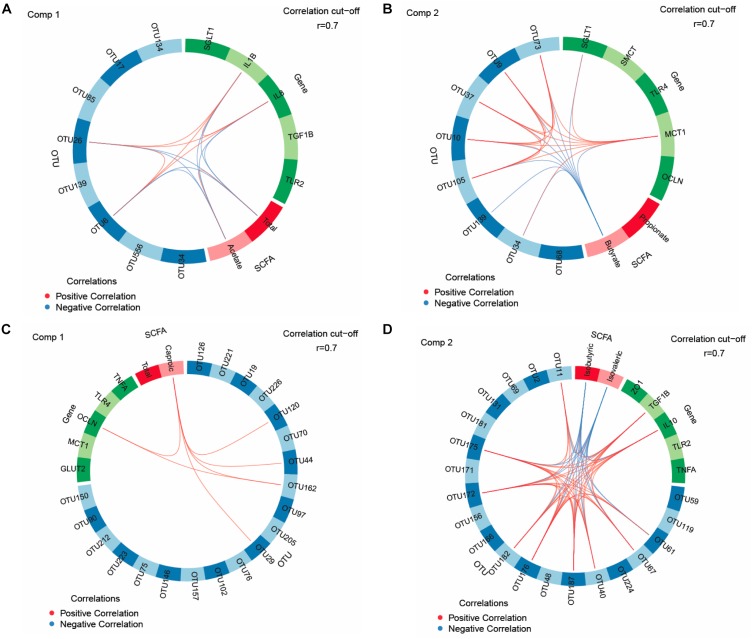
Circos plots of horizontal sparse partial least squares-discriminant analysis displaying correlations between the identified best discriminant operational taxonomic units (OTUs), short-chain fatty acids (SCFA, *n* = 5) and expression levels of target genes for component 1 and component 2 in the jejunum and ceca. Correlations for component 1 (comp) 1 **(A)** and 2 **(B)** in jejunum (OTUs, *n* = 8; SCFA, *n* = 2; genes, *n* = 5). Correlations for comp 1 **(C)** and 2 **(D)** in ceca (OTUs, *n* = 20; SCFA, *n* = 2; genes, *n* = 5). Positive and negative correlations (*| r*| > 0.7) are displayed by red and blue links, respectively. Relative abundance of bacterial OTUs > 0.01% of all reads. *GLUT2*, glucose transporter 2; *IL*, interleukin; *MCT1*, monocarboxylate transporter 1; *OCLN*, occludin; *SGLT1*, sodium-dependent glucose transporter 1; *SMCT*, sodium/monocarboxylate transporter; *TGF1B*, transforming growth factor beta-1; *TLR*, toll-like receptor; *TNFA*, tumor-necrosis factor alpha; *ZO1*, zonula occludens. The taxonomic identification of the most influential OTUs can be found in Supplementary Table S8.

**FIGURE 2:**
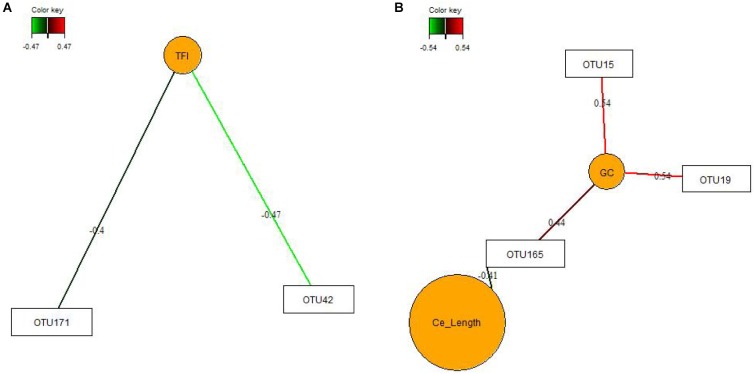
Covariations between the relative abundances of the most discriminant bacterial operational taxonomic units (OTU; relative abundances > 0.01%) and total feed intake (TFI) and total body weight gain (TBWG) **(A)** and cecal characteristics (organ size and histo-morphology; **B**). Associations were established using sparse partial least squares regression. Networks displayed graphically as nodes (OTUs and performance traits) and edges (biological relationship between nodes), with the edge color intensity indicating the level of the association: red, positive, and green, negative. Only the strongest pairwise associations are presented (*| r*| > 0.40). Ce_Length, average length of the ceca; GC, goblet cell counts.

We used sPLS-DA to identify relationships between the most discriminant OTUs, SCFAs and host mucosal expression of genes separately for the jejunum and ceca (Figure 1 and Supplementary Table S8). The sPLS-DA identified five *Lactobacillus*-OTUs (OTU17, OTU34, OTU85, OTU134, and OTU139) and one *Sphingomonas*-OTU (OTU556) as influential OTUs in the jejunum. However, they were not correlated to other discriminant features, whereas the [*Ruminocccus*]-like OTU (OTU26) and *Ruminococcaceae*-OTU (OTU6) negatively correlated to total SCFA and acetate and positively to expression levels of *IL1B* and *IL8* for component 1 (Figure 1A). For component 2, the 7 identified *Lactobacillus*-OTUs (OTU9, OTU10, OTU34, OTU37, OTU73, OTU105, and OTU139) all positively correlated with the jejunal expression levels of *MCT1* but negatively with the jejunal butyrate concentration (Figure 1B). The majority of identified most influential OTUs for component 1 and 2 in the ceca were *Clostridiales*- and *Ruminococcus*-OTUs (Figure 1C,D). For component 1 (Figure 1C), 2 *Clostridiales*-OTUs (OTU29 and OTU44) and 2 *Ruminococcus*-OTUs (OTU120 and OTU162) were positively associated with the cecal caproate concentration which in turn positively correlated to the cecal expression of *OCLN*. *Ruminococcus*-OTU162 also positively correlated to cecal *OCLN* expression for component 1. More positive and negative associations were detected in the ceca for component 2 (Figure 1D), showing that several *Clostridiales*-OTUs (OTU11, OTU40, OTU61, OTU67, OTU172, OTU175, OTU176, OTU182, and OTU187) were positively correlated to each other as well as to the cecal *TGF1B* and *IL10* expression and were negatively correlated to cecal isobutyrate and isovalerate. The branched-chain fatty acids were also negatively correlated to cecal expression levels of *TGF1B* and *IL10.* Relevance networking identified two *Clostridiales*-OTUs (OTU42 and OTU171) in cecal digesta that were negatively associated with TFI (*r* < −0.4; Figure 2A), but only weaker relationships for RFI and OTUs in the ceca were found (|*r*| < 0.33; data not shown). Moreover, relative networking showed positive relationships (*r* > 0.4) between cecal crypt depth and two *Anaerotruncus*-OTUs (OTU15 and OTU19) and the *Lachnospiraceae*-OTU165 (Figure 2B). The OTU165 was further negatively associated with the length of the ceca.

## Discussion

In using a FMT prepared from low RFI donor chickens, the present study aimed to influence the early bacterial colonization in order to modulate the “gut-brain axis” with the goal to improve chicken’s FE later in life. Albeit increasing the TFI and TBWG (Siegerstetter et al., 2018a), the FMT did not modify bird’s FE, suggesting persisting RFI-associated differences in host physiology and metabolism. Despite selecting chickens with extremely low and high RFI values when reaching market weight, only a few RFI-associated differences for the jejunal and cecal bacterial composition, and intestinal SCFA, physiology, structure and functioning were detected in the present study. Obviously, the intestine only played a moderate role for the present low and high RFI phenotypes. From the observed RFI-associated variation, energy-saving mechanisms and a potential attenuation of the mucosal immune response were indicated by the higher jejunal permeability, shorter villi in the ileum, and enhanced cecal expression of the anti-inflammatory cytokine *IL10* in low RFI chickens. Since we aimed to modulate the early colonization, effects of the FMT on the jejunal bacterial composition in 5-week old birds were generally expected to be small, as other factors such as the diet and feed intake become more important for species abundances than the FMT with increasing age (Siegerstetter et al., 2018b). Nevertheless, the bacteria found in the FMT formed the dominant bacterial taxa in the jejunal and cecal communities, including *Enterobacteriaceae, Turicibacter, Ruminococcaceae*, and *Lactobacillus*. Moreover, FMT-associated bacterial changes were evident in cecal digesta at 5 weeks of life, which may be due to chicken’s physiological peculiarity that the ceca form true blind sacks. FMT-related taxonomic changes indicated alterations in the cecal abundances of amylolytic, pullulanolytic and hemicellulolytic species belonging to the genera *Lactobacillus*, *Dorea*, and *Ruminococcus*. Moreover, results showed that there was a long-lasting effect of the FMT on the SCFA profile in the crop, as well as on duodenal and cecal development. Overall, relationships among the most influential OTUs, SCFA and expressed genes obtained from sPLS-DA support that certain bacteria, including *Ruminococcocaceae, Lactobacillus*, and unclassified *Clostridiales*, and SCFA in jejunal and cecal digesta modulated expression levels of cytokines, the tight-junction protein *OCLN* and nutrient transporters for glucose and SCFA uptake.

The crop was the intestinal site where bacterial activity was most affected by the FMT, whereas effects on the bacterial community and metabolites were small in jejunum and ileum. Without having analyzed compositional changes, results for the crop indicated marked alterations in fermentation pathways used by the microbes which may be due to differences in the early colonization of the crop after FMT administration, resulting in the FMT-related decrease in total SCFA concentration and altered SCFA profile. In general, the lower acetate level may be indicative of reduced carbohydrate fermentation, whereas increased levels of branched-chain fatty acids and valerate hinted at increased protein fermentation. Typically, the crop of chickens is colonized by lactic acid bacteria (Stanley et al., 2014). Due to the low proportion of *Lactobacillus*-OTUs in the FMT, the more dominant bacteria in the FMT, such as *Enterobacteriaceae* and *Turicibacter*, may have replaced lactic acid bacteria, thereby altering the SCFA profile. As the effects on SCFA observed in the crop vanished until the jejunum, it can be speculated whether the harsh conditions in gizzard and proventriculus or bacterial substrate availability may have prevented those species from establishing similar metabolic capacities in the jejunum. The anatomical peculiarity of the cecal blind sacks probably guaranteed that FMT residuals remained in the ceca, explaining why FMT-related shifts in the bacterial composition were identifiable in the 5-week old birds. It can be assumed that the FMT modified the maturational succession of bacteria, leading to the observed alterations in complex carbohydrate-degrading genera, e.g., the lower cecal abundance of *Lactobacillus* would support this assumption. Other starch-degrading bacteria may have taken over their intestinal niche, such as the unclassified *Lachnospiraceae* genus *Ruminococcus*, which were or tended to be increased in cecal digesta of birds receiving the FMT, respectively, and may have contributed to the higher cecal propionate concentration in the FMT group. Since the FMT enlarged the duodenum and led to structural and physiological alterations in the ceca, long-term alterations in the microbiota-host networking can be assumed. This would be supported by the current relevance networks for goblet cell counts, indicating that *Anaerotruncus* and *Lachnospiraceae* species may have stimulated the development of goblet cells. It may be predicted from the shallower crypts in the ceca of birds receiving the FMT that either taxonomic shifts or increased propionate concentration stimulated the cell renewal of the mucosal lining (Zhang et al., 2018).

The bacterial communities in jejunal and cecal digesta played only a minor role for the observed phenotypic variation in RFI. Relevance networks showed the importance of the TFI for the cecal abundance of two *Clostridiales*-OTUs but identified no strong relationship of bacteria with RFI. Results from the univariate analysis for the jejunal community supported our previous findings of an association of intestinal *Turicibacter* with high RFI, which can most likely be related to the increased feed intake in high RFI birds (Siegerstetter et al., 2018b). Whether this was also true for the trends for the increase in *Phenylobacterium* in jejunal digesta and an unclassified *Lachnospiraceae* genus in the ceca with high RFI or whether other changes in host physiology (e.g., mucin production) or cross-feeding of microbial metabolites are behind these RFI-associated variations can only be speculated. Results for SCFA, especially acetate, in jejunal digesta indicated RFI-associated variation in the bacterial metabolic activity, which may have been linked to jejunal abundances of *Ruminococcaceae*-OTUs according to the correlations in the circos plot for component 1. However, the FMT × RFI interaction indicated an interference of the FMT on early bacterial colonization as this low RFI-associated decrease in total SCFA was mainly detectable in animals receiving the FMT. This is interesting since the expression levels of the SCFA transporter *SMCT* were higher in low RFI chickens but only in the CT group, which is in accordance with previous results from our group and may point toward increased SCFA uptake if translated into functional protein (Metzler-Zebeli et al., 2018b). If true, this might explain the equal jejunal SCFA concentrations across RFI ranks in the CT group and the RFI-associated difference in jejunal SCFA in the FMT group. The trend of less jejunal acetate, in turn, may either suggest lower production or greater mucosal uptake of acetate in low RFI animals. In line with that, the greater mucosal-to-serosal flux of HRP (trend) and basal G_T_ suggested greater paracellular nutrient flux in the jejunum of low RFI birds. The greater change in G_T_ (ΔG_T_) as response to the glucose stimulation in low RFI chickens also let assume a greater glucose absorption capacity in low RFI chickens. Correlations among key features, as illustrated by the circos plot for component 2, further showed a positive link between *MCT1* and *SGLT1* expression at the jejunal mucosa, supporting an enhanced jejunal nutrient absorption in low RFI chickens. Without having measured lactate, the many positive associations with *Lactobacillus*-OTUs let speculate that lactate may have been the major fermentation product stimulating *MCT1* expression in the jejunal mucosa. Interestingly, an opposite physiological adaptation was indicated for SCFA uptake in the ceca where low RFI animals of the FMT group had a higher expression of *MCT1* compared to the high RFI animals but not in the CT group, again suggesting developmental changes in mucosal functioning due to the FMT. Other RFI-associated variation related to energy saving mechanisms (Metzler-Zebeli et al., 2018b) were the shorter jejunal villi and smaller villus surface in low RFI chickens, pointing toward lower maintenance needs for the renewal of the epithelial surface. In contrast, the heavier ceca in low versus high RFI birds may indicate more efficient microbial fiber utilization. The trend for a lower expression of *NFKB* at the jejunal mucosa and of *CLDN1* at the cecal mucosa in low versus high RFI animals may have indicated that fewer nutrients were diverted from growth toward the immune system in low RFI chickens (Broom and Kogut, 2017). As anti-inflammatory *IL10* was also greater expressed at the cecal mucosa of low RFI chickens, this may point toward an attenuation of the proinflammatory immune response. Moreover, present correlations for the jejunum and ceca support that differences in microbial signaling modified the jejunal and cecal expression of pro- and anti-inflammatory cytokines. To demonstrate this, *Ruminococcaceae*-OTUs (i.e., OTU6 and OTU26) may have stimulated proinflammatory *IL1B* and *IL8* expression at the jejunal mucosa, whereas acetate attenuated their expression; the latter possibly being mediated via mucosal G-protein activation (McKenzie et al., 2017). Especially, the positive relationships for component 2 showed that many of the most influential *Clostridiales*-OTUs in cecal digesta may have triggered a greater mucosal tolerance toward the commensal microbiota by increasing the expression levels of *IL10* and *TGFB1* at the cecal mucosa (Wahl et al., 2006).

In conclusion, results suggest that the intestine only played a moderate role for the RFI-associated variation of the present low and high RFI phenotypes and may be related to energy-saving mechanisms, improved nutrient absorption and moderation of the mucosal immune response. Albeit the high FE of donor chickens was not transferred with the FMT, modulating the early microbial colonization resulted in long-lasting changes in bacterial taxonomic (ceca) and metabolite (crop) composition as well as host intestinal development. From this point of view, the present study emphasizes FMT as a useful tool to modify intestinal fermentation, bacterial composition and microbe-host signaling in chickens. However, effects of the FMT were mostly independent from those of the RFI-associated variation in intestinal physiology and function, supporting the importance of host-specific factors for the observed RFI-associated variation.

## Data Availability

The datasets generated for this study can be accessed from NCBI’s BioProject SRA database, PRJNA392215 and PRJNA529338.

## Ethics Statement

This study was conducted at the Institute of Animal Nutrition and Functional Plant Compounds, University of Veterinary Medicine Vienna, Austria. The animal procedures were approved by the Institutional Ethics Committee of the University of Veterinary Medicine Vienna and the Austrian National Authority according to paragraph 26 of Law for Animal Experiments, Tierversuchsgesetz 2012 – TVG 2012 (GZ 68.205/0148-II/3b/2015).

## Author Contributions

BM-Z, EM, PL, and NO conceived and designed the experiments. S-CS and BM-Z performed the experiments. BM-Z analyzed the data, wrote the statistical codes, interpreted the data, drafted the manuscript, and took primary responsibility for the final content. S-CS, PL, QZ, EM, and NO revised the manuscript. All authors read and approved the final manuscript.

## Conflict of Interest Statement

The authors declare that the research was conducted in the absence of any commercial or financial relationships that could be construed as a potential conflict of interest.

## Footnotes

1 https://cutadapt.readthedocs.org/

2 http://ccb.jhu.edu/software/FLASH/
