# Supplementary material for: Fecal Microbiota Transplant From Highly Feed Efficient Donors Affects Cecal Physiology and Microbiota in Low- and High-Feed Efficient Chickens
Source: Front Microbiol. 2019 Jul 9;10:1576. doi: 10.3389/fmicb.2019.01576 (PMC6629952; doi:10.3389/fmicb.2019.01576)
Supplement: Supplementary file 1 [file Data_Sheet_1.PDF]

Running Title: Fecal microbiota transplant and gut functions

Fecal microbiota transplant from highly feed efficient donors affects cecal physiology and microbiota in low- and high-feed efficient chickens

Barbara U. Metzler-Zebeli[1], Sina-Catherine Siegerstetter[1], Elizabeth Magowan[2], Peadar G. Lawlor[3], Niamh E. O´Connell[4], Qendrim Zebeli[1]

[1]Institute of Animal Nutrition and Functional Plant Compounds, Department for Farm Animals and Veterinary Public Health, University of Veterinary Medicine Vienna, Vienna, Austria.
[2]Agri-Food and Biosciences Institute, Agriculture Branch, Hillsborough, Northern Ireland, UK.
[3]Teagasc, Pig Development Department, Animal & Grassland Research & Innovation Centre, Moorepark, Ireland.
[4]Institute for Global Food Security, Queen´s University Belfast, Northern Ireland, UK.

***Correspondence:**
Dr. Barbara Metzler-Zebeli
barbara.metzler@vetmeduni.ac.at

Table S1 Total feed intake (TFI), total body weight gain (TBWG), and residual feed intake (RFI) values of low and high RFI broiler chickens receiving either a fecal microbiota transplant (FMT) or a control transplant (CT)[1]

	FMT		CT			*P* value		
Item[a]	Low RFI	High RFI	Low RFI	High RFI	SEM	FMT	RFI	FMT×RFI
TFI (g)	2315	2631	2327	2568	57.3	0.665	<0.001	0.517
TBWG (g)	1641	1654	1656	1640	39.8	0.980	0.964	0.724
RFI (g)	-108	198	-117	155	25.2	0.310	<0.001	0.506

Data are presented as least-square means and pooled SEM. Low RFI FMT females, $n = 8$; low RFI FMT males, $n = 7$; high RFI FMT females, $n = 7$; high RFI FMT males, $n = 6$; low RFI CT females, $n = 7$; low RFI CT males, $n = 7$; high RFI CT females, $n = 7$; high RFI CT males, $n = 7$.

[a]TFI, TBWG and RFI were calculated for the experimental period from 9 to 30 days post-hatch.

Sex affected TFI, TBWG ($P < 0.001$) and RFI ($P < 0.05$).

Table S2 Alpha-diversity of bacterial microbiota communities in ileal and cecal digesta of low and high residual feed intake (RFI) broiler chickens receiving either a fecal microbiota transplant (FMT) or a control transplant (CT)[1]

	FMT		CT			P value		
Index	low RFI	high RFI	low RFI	high RFI	SEM	FMT	RFI	FMT×RFI
Jejunum								
Observed OTUs	102	90	83	106	14.11	0.913	0.685	0.214
Simpson	0.75	0.73	0.68	0.72	0.050	0.475	0.788	0.543
Shannon	2.26	2.14	1.85	2.10	0.201	0.266	0.759	0.358
Ceca								
Observed OTUs	314	312	336	338	31.76	0.458	0.997	0.944
Simpson	0.94	0.93	0.94	0.93	0.010	0.663	0.528	0.878
Shannon	3.77	3.76	3.87	3.79	0.090	0.420	0.615	0.699

[1]Data are presented as least-square means and pooled SEM. Low RFI FMT females, *n* = 8; low RFI FMT males, *n* = 7; high RFI FMT females, *n* = 7; high-RFI FMT males, *n* = 6; low RFI CT females, *n* = 7; low-RFI CT males, *n* = 7; high-RFI CT females, *n* = 7; high-RFI CT males, *n* = 7.

Table S3 Relative abundance of bacterial phyla (% of all reads) present in jejunal and cecal digesta of low and high residual feed intake (RFI) broiler chickens receiving either a fecal microbiota transplant (FMT) or a control transplant (CT)[1]

	FMT		CT			P value		
Phyla	low RFI	high RFI	low RFI	high RFI	SEM	FMT	RFI	FMT×RFI
Jejunum								
Firmicutes	59.1	51.3	70.7	59.6	8.030	0.222	0.245	0.838
Proteobacteria	32.6	39.0	24.7	34.6	8.200	0.457	0.328	0.833
Cyanobacteria	7.5	9.1	4.3	5.2	3.073	0.258	0.688	0.902
Tenericutes	0.20	0.01	0.04	0.04	0.063	0.290	0.137	0.135
Actinobacteria	0.31	0.48	0.11	0.48	0.169	0.542	0.118	0.540
Other	0.28	0.12	0.10	0.13	0.120	0.492	0.567	0.418
Ceca								
Firmicutes	92.4	93.5	95.1	92.3	2.510	0.764	0.730	0.441
Proteobacteria	6.6	5.2	3.3	6.9	2.500	0.769	0.665	0.318
Tenericutes	0.68	1.01	1.15	0.40	0.264	0.788	0.429	0.045
Actinobacteria	0.09	0.15	0.11	0.12	0.025	0.892	0.209	0.302
Other	0.21	0.17	0.27	0.23	0.034	0.096	0.225	0.980

[1]Data are presented as least-square means and pooled SEM. Low RFI FMT females, n = 8; low RFI FMT males, n = 7; high RFI FMT females, n = 7; high-RFI FMT males, n = 6; low RFI CT females, n = 7; low-RFI CT males, n = 7; high-RFI CT females, n = 7; high-RFI CT males, n = 7.

Table S4 Relative abundance of most abundant bacterial genera (>0.1% of all reads) present in jejunal and cecal digesta of low and high residual feed intake (RFI) broiler chickens receiving either a fecal microbiota transplant (FMT) or a control transplant (CT)[1]

	FMT		CT			P value		
Genus	low RFI	high RFI	low RFI	high RFI	SEM	FMT	RFI	FMT×RFI
Jejunum								
o__*Clostridiales*;f__;g__	2.38	1.68	0.86	1.75	0.730	0.324	0.895	0.281
f__*Enterobacteriaceae*;g__	28.92	34.74	23.62	31.90	8.528	0.635	0.413	0.886
g__*Lactobacillus*	38.58	31.79	55.42	38.37	9.614	0.229	0.221	0.596
f__*Ruminococcaceae*;g__	3.02	0.85	0.68	0.94	0.701	0.116	0.178	0.088
g__*Turicibacter*	3.79	8.49	4.51	15.09	4.076	0.374	0.067	0.474
o__*Streptophyta*;f__;g__	7.46	9.08	4.32	5.18	3.073	0.258	0.688	0.902
f__*Peptostreptococcaceae*;g__	7.14	3.94	7.41	0.31	3.780	0.658	0.179	0.609
g__*Anaerotruncus*	0.20	0.075	0.034	0.12	0.082	0.482	0.844	0.206
g__*Ruminococcus*	0.17	0.038	0.098	0.095	0.062	0.902	0.283	0.300
f__*Lachnospiraceae*;g__	0.32	0.092	0.13	0.15	0.088	0.449	0.260	0.148
g__[*Ruminococcus*]	0.52	0.066	0.040	0.10	0.166	0.190	0.245	0.128
g__*Oscillospira*	0.26	0.084	0.078	0.051	0.076	0.168	0.191	0.341
g__*Bacillus*	0.22	0.39	0.14	0.10	0.191	0.339	0.721	0.584
g__*Enterococcus*	1.42	1.41	0.42	1.62	0.633	0.534	0.352	0.346
o__*RF39*;f__;g__	0.21	0.015	0.042	0.043	0.062	0.290	0.137	0.135
g__*Coprococcus*	0.054	0.076	0.014	0.026	0.035	0.197	0.630	0.879
g__*Blautia*	0.080	0.058	0.021	0.031	0.024	0.086	0.806	0.507
o__*Rickettsiales*;f__;g__	0.92	0.75	0.55	0.50	0.322	0.345	0.743	0.860
o__*Clostridiales*;f__;g__	0.23	0.79	0.72	0.21	0.386	0.911	0.952	0.174
g__*Acinetobacter*	1.18	0.65	0.015	0.38	0.600	0.239	0.892	0.459
g__*Sphingomonas*	0.18	0.58	0.041	0.24	0.173	0.175	0.090	0.561
f__*0319-6G20*;g__	0.16	0.53	0.042	0.35	0.199	0.455	0.092	0.873
f__*Christensenellaceae*;g__	0.012	0.014	0	0.003	0.008	0.163	0.764	0.955
g__*Phenylobacterium*	0.17	0.48	0.036	0.15	0.118	0.053	0.075	0.384
g__*Dorea*	0.020	0.10	0.013	0.017	0.037	0.209	0.239	0.288
g__*Lactococcus*	0.049	0.81	0.010	0.056	0.274	0.153	0.146	0.196
f__*Clostridiaceae*;g__	0.068	0.050	0.022	0.060	0.041	0.655	0.814	0.505
g__*Clostridium*	0.43	0.20	0.065	0.068	0.211	0.256	0.596	0.585
f__*Lachnospiraceae*;f__;g__	0.020	0.001	0.010	0.030	0.011	0.400	0.965	0.073
g__*Proteus*	0.13	0.080	0.002	0.007	0.061	0.107	0.716	0.659
Ceca								
o__*Clostridiales*;f__;g__	57.30	56.40	62.10	57.32	3.919	0.469	0.473	0.623
f__*Enterobacteriaceae*;g__	3.33	5.12	3.08	6.92	1.897	0.685	0.144	0.591
g__*Lactobacillus*	0.68	0.35	1.23	0.90	0.254	0.036	0.195	0.995
f__*Ruminococcaceae*;g__	17.63	16.60	14.03	16.41	1.853	0.312	0.718	0.363
g__*Turicibacter*	0.70	0.95	0.69	0.84	0.320	0.863	0.540	0.879
f__*Peptostreptococcaceae*;g__	0.02	0.02	0.01	0.02	0.006	0.320	0.550	0.902
g__*Anaerotruncus*	2.67	4.19	4.04	4.13	1.508	0.668	0.597	0.637
g__*Ruminococcus*	3.11	3.70	2.57	2.94	0.380	0.093	0.215	0.772
f__*Lachnospiraceae*;g__	2.39	2.55	1.87	2.18	0.317	0.167	0.449	0.813

g__[*Ruminococcus*]	2.42	2.79	1.30	1.79	0.460	0.025	0.352	0.904
g__*Oscillospira*	2.06	2.16	1.88	2.04	0.199	0.458	0.524	0.874
g__*Bacillus*	0.34	0.61	2.68	0.81	0.927	0.176	0.392	0.254
g__*Enterococcus*	0.075	0.076	0.087	0.079	0.034	0.834	0.913	0.908
o__*RF39*;f__;g__	0.68	1.01	1.15	0.40	0.264	0.788	0.429	0.045
g__*Coprococcus*	0.89	0.76	0.74	0.82	0.107	0.708	0.835	0.345
g__*Blautia*	0.75	0.92	0.67	0.79	0.141	0.478	0.308	0.856
o__*Clostridiales*;f__;g__	0.15	0.10	0.09	0.10	0.027	0.341	0.500	0.327
g__*Sphingomonas*	1.00	0.001	0.002	0.003	0.500	0.322	0.322	0.321
f__*0319*-6G20;g__	0.67	0.001	0.001	0	0.333	0.322	0.322	0.323
f__*Christensenellaceae*;g__	0.37	0.44	0.42	0.44	0.106	0.825	0.693	0.781
g__*Phenylobacterium*	0.62	0.001	0.001	0.001	0.310	0.322	0.322	0.322
g__*Dorea*	0.27	0.24	0.16	0.18	0.042	0.047	0.912	0.528
g__*Clostridium*	0.027	0.032	0.027	0.038	0.006	0.585	0.165	0.674
f__*Lachnospiraceae*;f__;g__	0.18	0.27	0.15	0.21	0.035	0.190	0.032	0.520
g__*Proteus*	0.12	0.037	0.25	0.016	0.129	0.661	0.230	0.549
f__*Dehalobacteriaceae*;g__	0.13	0.13	0.10	0.10	0.028	0.313	0.941	0.990
f__*Bacillaceae*;g__	0.016	0.022	0.098	0.035	0.023	0.050	0.229	0.146
g__*Slackia*	0.041	0.084	0.051	0.061	0.020	0.743	0.184	0.405
f__*Erysipelotrichaceae*;g__	0.044	0.061	0.041	0.033	0.010	0.114	0.664	0.220
g__*Coprobacillus*	0.033	0.074	0.031	0.027	0.018	0.162	0.305	0.203
f__*Coriobacteriaceae*;g__	0.025	0.037	0.040	0.032	0.007	0.473	0.770	0.139
f__*Ruminococcaceae*;g__	0.030	0.027	0.048	0.049	0.008	0.012	0.889	0.833
g__*Eggerthella*	0.022	0.024	0.019	0.024	0.005	0.755	0.506	0.819
f__[*Mogibacteriaceae*];g__	0.012	0.009	0.010	0.017	0.004	0.490	0.541	0.224

[1]Data are presented as least-square means and pooled SEM. Low RFI FMT females, *n* = 8; low RFI FMT males, *n* = 7; high RFI FMT females, *n* = 7; high-RFI FMT males, *n* = 6; low RFI CT females, *n* = 7; low-RFI CT males, *n* = 7; high-RFI CT females, *n* = 7; high-RFI CT males, *n* = 7.

Table S5 Differences in the concentration of total short-chain fatty acids (SCFA) and molar proportions (%) of the individual SCFA in crop, jejunal, ileal and cecal digesta of low and high residual feed intake (RFI) broiler chickens receiving either a fecal microbiota transplant (FMT) or a control transplant (CT)[1]

	FMT		Control			P value		
Item	Low RFI	High RFI	Low RFI	High RFI	SEM	FMT	RFI	FMT × RFI
Crop								
Total SCFA (μmol/g)	43.1	44.7	60.2	59.6	7.91	0.052	0.955	0.885
Molar proportion (%)								
Acetate	89.1	87.2	90.3	89.4	1.163	0.152	0.249	0.658
Propionate	1.73	2.39	0.99	0.67	0.416	0.006	0.686	0.250
Isobutyrate	6.89	6.81	7.06	8.64	1.003	0.329	0.458	0.412
Butyrate	1.08	1.48	0.93	0.88	0.198	0.068	0.383	0.259
Isovalerate	0.20	0.33	0.04	0.01	0.081	0.005	0.544	0.353
Valerate	0.33	0.64	0.19	0.10	0.133	0.017	0.427	0.136
Caproate	0.72	1.18	0.55	0.30	0.157	0.002	0.486	0.029
Jejunum								
Total SCFA (μmol/g)	47.9[b]	61.7[a]	55.0[ab]	54.7[ab]	3.24	0.994	0.041	0.036
Molar proportion (%)								
Acetate	89.74	89.14	88.26	88.06	0.844	0.144	0.635	0.812
Propionate	0.55	0.48	0.53	0.40	0.099	0.635	0.350	0.746
Isobutyrate	8.48	9.31	10.34	10.65	0.791	0.055	0.472	0.737
Butyrate	0.31	0.33	0.27	0.22	0.076	0.335	0.854	0.693
Isovalerate	0.47	0.42	0.37	0.40	0.142	0.682	0.957	0.804
Valerate	0.03	0.02	0.02	0.01	0.021	0.535	0.602	0.865
Caproate	0.43	0.28	0.21	0.26	0.100	0.259	0.655	0.343
Ileum								
Total SCFA (μmol/g)	41.2	44.9	38.5	38.8	3.06	0.158	0.514	0.586
Molar proportion (%)								
Acetate	91.62	91.38	91.83	91.24	0.471	0.942	0.382	0.715
Propionate	0.79	0.81	0.78	0.85	0.122	0.880	0.700	0.862
Isobutyrate	6.94	7.05	6.73	7.23	0.358	0.958	0.396	0.592
Butyrate	0.07	0.06	0.13	0.07	0.053	0.557	0.459	0.604
Isovalerate	0.55	0.55	0.35	0.50	0.122	0.325	0.559	0.567
Valerate	.	.	.	.	.	.	.	.
Caproate	0.03	0.14	0.17	0.12	0.079	0.481	0.708	0.292
Ceca								
Total SCFA (μmol/g)	147.0	155.9	126.6	139.1	13.71	0.186	0.460	0.896
Molar proportion (%)								
Acetate	80.57	82.12	81.88	81.58	1.795	0.830	0.740	0.609
Propionate	5.15	6.02	4.65	4.34	0.515	0.044	0.607	0.266
Isobutyrate	0.75	0.54	0.93	1.00	0.173	0.081	0.710	0.430
Butyrate	11.40	9.50	10.27	10.16	1.724	0.891	0.579	0.607
Isovalerate	0.70	0.52	0.80	1.07	0.192	0.106	0.816	0.263

Valerate	1.06	0.90	1.10	1.20	0.148	0.263	0.839	0.381
Caproate	0.37	0.39	0.38	0.66	0.110	0.231	0.190	0.262

[1]Data are presented as least-square means and pooled SEM. Low RFI FMT females, *n* = 8; low RFI FMT males, *n* = 7; high RFI FMT females, *n* = 7; high-RFI FMT males, *n* = 6; low RFI CT females, *n* = 7; low-RFI CT males, *n* = 7; high-RFI CT females, *n* = 7; high-RFI CT males, *n* = 7. ND, not detected.

[a,b]Different superscripts within a row indicate significant difference ($P \leq 0.05$).

Table S6 Differences in visceral organ size of low and high residual feed intake (RFI) broiler chickens receiving either a fecal microbiota transplant (FMT) or a control transplant (CT)[1]

	FMT		CT			P value		
Item	low RFI	high RFI	low RFI	high RFI	SEM	FMT	RFI	FMT × RFI
Body weight (BW) at sampling (kg)	2.38	2.38	2.43	2.44	0.070	0.432	0.968	0.974
Length (cm/kg BW)								
Duodenum	12.6	12.3	11.5	11.5	0.40	0.024	0.630	0.693
Jejunum	42.3	41.6	41.5	39.3	1.44	0.282	0.307	0.629
Ileum	13.3	12.9	12.6	13.1	0.55	0.622	1.000	0.402
Ceca (average of the two)	6.4	6.6	6.3	5.8	0.23	0.061	0.485	0.168
Colon	3.1	3.2	3.2	3.1	0.14	0.952	0.746	0.346
Total gut	84.2	83.1	81.4	78.6	2.20	0.100	0.376	0.695
Weight (g/kg BW)								
Crop	3.1	3.2	3.2	3.0	0.30	0.864	0.812	0.737
Proventriculus	4.1	4.1	4.1	4.4	0.19	0.565	0.412	0.450
Gizzard	12.5	14.6	13.4	13.0	0.63	0.571	0.203	0.054
Duodenum	5.0	4.9	4.8	4.8	0.22	0.527	0.740	0.809
Jejunum	16.1	17.0	15.8	16.2	0.54	0.300	0.216	0.693
Ileum	2.5	2.8	2.7	2.7	0.14	0.769	0.357	0.243
Ceca (average of the two)	1.1	1.0	1.1	1.0	0.04	0.618	0.027	0.864
Colon	1.3	1.3	1.3	1.3	0.07	0.844	0.648	0.711
Total gut	45.8	48.9	46.4	46.4	1.02	0.348	0.143	0.134
Pancreas	1.9	1.9	1.7	1.8	0.06	0.188	0.663	0.527
Liver	21.2	21.1	21.5	20.8	0.70	0.974	0.503	0.648
Heart	5.5	5.7	5.1	5.5	0.23	0.205	0.157	0.819

[1]Data are presented as least-square means and pooled SEM. Low RFI FMT females, n = 8; low RFI FMT males, n = 7; high RFI FMT females, n = 7; high-RFI FMT males, n = 6; low RFI CT females, n = 7; low-RFI CT males, n = 7; high-RFI CT females, n = 7; high-RFI CT males, n = 7.

Table S7 Differences in intestinal histo-morphology of low and high residual feed intake (RFI) broiler chickens receiving either a fecal microbiota transplant (FMT) or a control transplant (CT)[1]

	FMT		CT			P value		
Item	low RFI	high RFI	low RFI	high RFI	SEM	FMT	RFI	FMT×RFI
Jejunum								
Villus height (µm)	652.1	711.0	711.3	701.3	21.40	0.252	0.259	0.114
Villus width (µm)	47.7	47.9	48.0	48.4	1.11	0.746	0.803	0.969
Crypt depth (µm)	70.7	71.5	68.5	68.7	2.79	0.370	0.855	0.914
Villus surface	0.10	0.11	0.11	0.11	0.004	0.278	0.381	0.238
Villus height : crypt depth	9.9	10.5	10.9	10.7	0.38	0.118	0.619	0.283
Circular muscle (µm)	86.0	94.4	89.1	94.7	6.04	0.787	0.254	0.820
Longitudinal muscle (µm)	33.9	35.7	36.7	37.4	2.50	0.374	0.619	0.839
Goblet cells (counts /villus-crypt unit)	55.4	64.2	60.1	63.7	3.75	0.582	0.105	0.490
Lymphocytes (counts/villus-crypt unit)	12.9	15.3	13.9	14.1	1.25	0.953	0.294	0.403
Ileum								
Villus height (µm)	514.6	572.5	542.1	581.5	21.00	0.388	0.025	0.661
Villus width (µm)	51.7	53.1	51.8	52.2	1.38	0.770	0.504	0.723
Crypt depth (µm)	70.6	70.2	71.6	70.9	2.39	0.724	0.842	0.952
Villus surface	0.08	0.10	0.09	0.10	0.005	0.625	0.045	0.602
Villus height : crypt depth	7.4	8.3	7.7	8.3	0.39	0.693	0.081	0.722
Circular muscle (µm)	133.1	126.1	124.5	136.3	11.50	0.943	0.838	0.416
Longitudinal muscle (µm)	43.4	42.6	42.6	42.2	3.11	0.855	0.847	0.952
Goblet cells (counts /villus-crypt unit)	51.1	54.0	51.0	53.4	3.38	0.923	0.446	0.934
Lymphocytes (counts/villus-crypt unit)	13.6	15.6	13.5	13.8	1.56	0.559	0.476	0.589
Ceca								
Crypt depth (µm)	184.1	172.1	208.2	209.3	10.76	0.006	0.614	0.545
Circular muscle (µm)	200.8	206.1	232.3	218.2	11.38	0.062	0.697	0.398
Longitudinal muscle (µm)	50.2	57.9	55.8	52.3	3.38	0.997	0.539	0.107

Goblet cells (counts /villus-crypt unit)	3.5	3.5	4.7	4.6	0.60	0.068	0.958	0.943
Lymphocytes (counts/villus-crypt unit)	0.9	0.9	1.0	1.1	0.12	0.160	0.591	0.489

[1]Data are presented as least-square means and pooled SEM. Low RFI FMT females, *n* = 8; low RFI FMT males, *n* = 7; high RFI FMT females, *n* = 7; high-RFI FMT males, *n* = 6; low RFI CT females, *n* = 7; low-RFI CT males, *n* = 7; high-RFI CT females, *n* = 7; high-RFI CT males, *n* = 7.

Table S8 Taxonomy of most influential bacterial operational taxonomic units (OTU) identified using sparse partial least squares-discriminant analysis and presented in the circos plots

Item	Taxonomy	Item	Taxonomy
Jejunum		Jejunum	
Component 1		Component 2	
OTU6	*Ruminococcaceae*	OTU9	*Lactobacillus*
OTU17	*Lactobacillus*	OTU10	*Lactobacillus*
OTU26	*[Ruminococcus]*	OTU34	*Lactobacillus*
OTU34	*Lactobacillus*	OTU37	*Lactobacillus*
OTU85	*Lactobacillus*	OTU68	*Enterococcus cecorum*
OTU134	*Lactobacillus*	OTU73	*Lactobacillus*
OTU139	*Lactobacillus*	OTU105	*Lactobacillus*
OTU556	*Sphingomonas*	OTU139	*Lactobacillus*
Ceca		Ceca	
Component 1		Component 2	
OTU19	*Anaerotruncus*	OTU11	*Clostridiales*
OTU29	*Clostridiales*	OTU2	*Clostridiales*
OTU44	*Clostridiales*	OTU69	*Ruminococcaceae*
OTU70	*Ruminococcus*	OTU131	*Ruminococcaceae*
OTU75	*Clostridiales*	OTU181	*Clostridiales*
OTU76	*Oscillospira*	OTU175	*Clostridiales*
OTU90	*Clostridiales*	OTU171	*Clostridiales*
OTU97	*Clostridiales*	OTU172	*Clostridiales*
OTU102	*Clostridiales*	OTU156	*Clostridiales*
OTU120	*Ruminococcus*	OTU166	*Clostridiales*
OTU126	*Oscillospira*	OTU182	*Clostridiales*
OTU146	*Clostridiales*	OTU176	*Clostridiales*
OTU150	*Clostridiales*	OTU48	*RF39*
OTU157	*Ruminococcaceae*	OTU187	*Clostridiales*
OTU162	*Ruminococcus*	OTU40	*Clostridiales*
OTU205	*Ruminococcus*	OTU224	*Clostridiales*
OTU212	*Clostridiales*	OTU67	*Clostridiales*
OTU221	*Ruminococcaceae*	OTU61	*Clostridiales*
OTU223	*Clostridiales*	OTU119	*Oscillospira*
OTU226	*Ruminococcus*	OTU59	*Clostridiales*

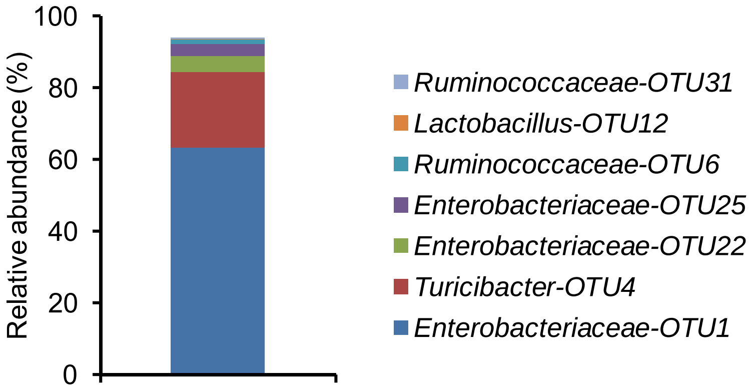

Figure S1 Taxonomic composition of fecal microbiota transplant (FMT). Mean values (*n* = 8; FMT inoculum of the three individual inoculation days of the two batches and pooled samples of the FMT inocula across the three inoculation days per batch). OTU, operational taxonomic unit. Only the most abundant OTUs are presented (relative abundance >0.1%).

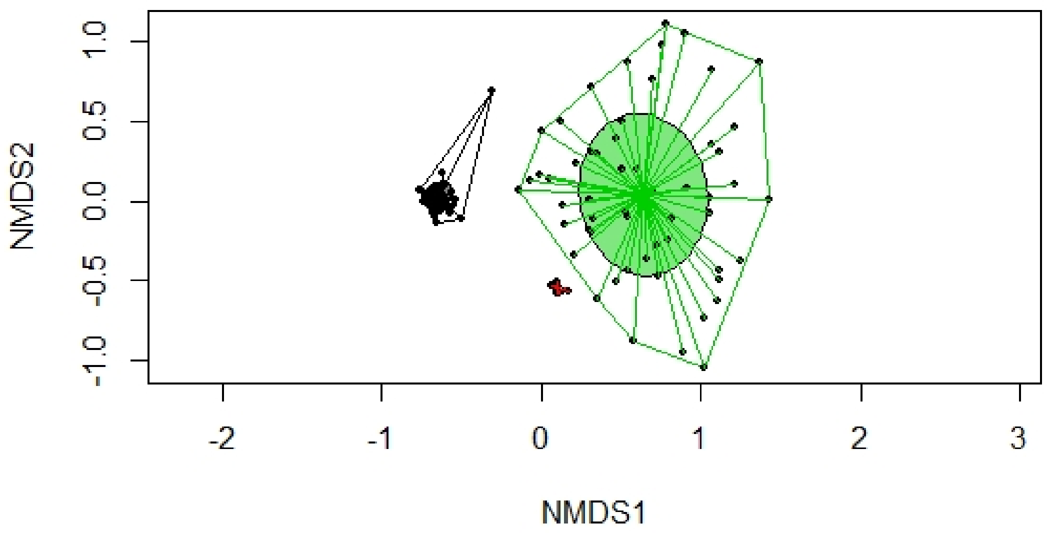

Figure S2 Nonmetric multidimensional scaling (NMDS) plot of pairwise Bray-Curtis dissimilarities between bacterial communities in jejunal (green) and cecal digesta (grey) and fecal microbiota transplant (red) (> 0.01 % relative abundance).
